# Multisample lipidomic profiles of irritable bowel syndrome and irritable bowel syndrome-like symptoms in patients with inflammatory bowel disease: new insight into the recognition of the same symptoms in different diseases

**DOI:** 10.1007/s00535-024-02148-1

**Published:** 2024-09-10

**Authors:** Guorong Chen, Xuan Wu, Huiting Zhu, Kemin Li, Junhai Zhang, Shijie Sun, Huifen Wang, Miao Wang, Bing Shao, Hui Li, Yanli Zhang, Shiyu Du

**Affiliations:** 1 Department of Gastroenterology, China-Japan Friendship Hospital(Institute of Clinical Medical Sciences), Peking Union Medical College, Chinese Academy of Medical Sciences, Beijing, 100029 China; 2 School of Public Health, Capital Medical University, Beijing, 100069 China; 3 Beijing Key Laboratory of Diagnostic and Traceability Technologies for Food Poisoning, Beijing Center for Disease Prevention and Control, Beijing, 100013 China; 4 Department of Gastroenterology, China-Japan Friendship Hospital, Beijing, 100029 China

**Keywords:** Irritable bowel syndrome, Inflammatory bowel disease, Lipidomics, Fatty acids

## Abstract

**Background:**

Overlapping clinical manifestations of irritable bowel syndrome (IBS) and IBS-like symptoms in patients with inflammatory bowel disease (IBD-IBS) present challenges in diagnosis and management. Both conditions are associated with alterations in metabolites, but few studies have described the lipid profiles. Our aim was to pinpoint specific lipids that contribute to the pathogenesis of IBS and IBD-IBS by analyzing multiple biologic samples.

**Methods:**

Diarrhea-predominant IBS (IBS-D) patients (*n* = 39), ulcerative colitis in remission with IBS-like symptoms patients (UCR-IBS) (*n* = 21), and healthy volunteers (*n* = 35) were recruited. IBS-D patients meet the Rome IV diagnostic criteria, and UCR-IBS patients matched mayo scores ≤ two points and Rome IV diagnostic criteria. Serum, feces, and mucosa were collected for further analysis. Lipid extraction was carried out by ultra-performance liquid chromatography-high resolution mass spectrometry (UPLC-HRMS).

**Results:**

Lipidomics of mucosa and serum samples significantly differed among the three groups. Feces showed the most altered lipid species, and the enrichment analysis of 347 differentially abundant metabolites via KEGG pathway analysis revealed that alpha-linolenic acid metabolism was significantly altered in the two groups (*P* < 0.01). The ratio of omega-6/omega-3 fatty acid were imbalance in serum samples.

**Conclusions:**

This study revealed a comprehensive lipid composition pattern between IBS-D patients and UCR-IBS patients. We found several distinctive lipids involved in alpha-linolenic acid metabolism, reflecting an imbalance in the omega-6/omega-3 fatty acid ratio. Compared to mucosa and serum samples, fecal samples might have more advantages in lipidomics studies due to the convenience of sample collection and effectiveness in reflecting metabolic information.

**Supplementary Information:**

The online version contains supplementary material available at 10.1007/s00535-024-02148-1.

## Introduction

Irritable bowel syndrome (IBS) is one of the most common functional gastrointestinal disorders, with a prevalence of approximately 11% in Western countries and 2–10% in China [1–3]. Another common bowel disease, inflammatory bowel disease (IBD), is receiving increasing attention, especially in developing countries. With economic development and society becoming more western, the incidence of IBD is also increasing, and the healthcare medical burden and concerns have increased [4, 5]. Although IBS and IBD belong to two distinct disease categories, it has been noted that IBD patients, especially during the remission period, develop IBS-like symptoms, which we refer to as IBD-IBS [6, 7]. In addition to the overlapping clinical symptoms, the pathogenesis and influencing factors of these conditions share many commonalities, such as intestinal flora, brain-gut axis regulation, and psychiatric factors [8–10]. Studies have shown that organic gastrointestinal disease occurs after the diagnosis of IBS and is associated with a significantly increased risk of developing IBD, indicating that there is an underlying intrinsic association between the two diseases [11, 12]. However, it is difficult to accurately analyze and characterize these two disorders by relying only on traditional testing methods and clinical experiences, and new technologies, especially new biomarkers, are urgently needed to improve the understanding of the pathophysiology and provide better treatment decisions.

Lipids play a very important role in the human body. As a major component of the cell membrane, lipids are widely involved in cell signaling and barrier regulation and are also important precursors of inflammatory and anti-inflammatory mediators [13]. Through lipidome analysis, specific lipid molecular patterns can be identified to help diagnose and understand diseases earlier and more accurately [14, 15]. Our previous study revealed that the levels of medium-chain and long-chain fatty acids in colon mucosa biopsies were able to distinguish IBS-D patients from healthy people [16]. Diab et al. [17] performed lipidomic analysis of the intestinal mucosa of ulcerative colitis (UC) patients and reported significant changes in the levels of phosphatidylcholine (PC), ceramide (Cer), and sphingomyelin (SM), suggesting that these lipids might be involved in the inflammatory response in UC. Analysis of urine samples revealed that the level of phosphatidyl choline acyl-alkyl C38:6 in IBS patients was significantly lower than that in healthy control individuals, suggesting a unique urinary metabolome profile in IBS patients [18]. Nonetheless, most of these studies have conducted their analyses based only on a single sample type, but certain metabolites may be enriched in different biologic samples, thus resulting in omissions and deviations. By combining multiple biologic samples, comprehensive metabolic information can be obtained, contributing to an in-depth understanding of metabolic activities and changes under different conditions [19, 20].

In this study, we conducted multiple-sample integrated lipidomic analysis of serum, feces, and intestinal mucosal tissues from IBS and IBD-IBS patients using liquid chromatography‒mass spectrometry (LC‒MS/MS) and bioinformatics. This study aimed to obtain a comprehensive lipidomic profile, further investigating the important lipid metabolites in both the IBS and IBD-IBS groups, and identify the changes in specific lipid molecules in different biologic samples and groups.

## Methods

### Study design

This single-center, case‒control study was conducted from September 2022 to August 2023 in the Department of Gastroenterology in China-Japan Friendship Hospital, Beijing, China.

### Chemicals and reagents

LC‒MS grade acetonitrile and methanol were purchased from Honeywell (USA). LC‒MS grade isopropanol, LC grade methyl tert-butyl ether (MTBE), formic acid and ammonium acetate were obtained from Fisher (Thermo Fisher™ Chemical, Waltham, MA, USA). Ultrapure water was purified by a Milli‒Q gradient system (Millipore, Milford, MA, USA).

### Participants

This study included three groups: the health control (HC) group, the diarrhea-predominant IBS (IBS-D) group, and the ulcerative colitis patients in remission with IBS-like symptoms (UCR-IBS) group. All participants were aged 18–65 years with a level of fecal calprotectin less than 50 mg/L. Briefly, patients in the IBS-D group met the Rome IV diagnostic criteria. UCR-IBS group should meet the following criteria: ① Diagnosed with mild ulcerative colitis, with mesalazine used as the remission medication; ② Have colonoscopy within a year; ③ Mayo score ≤ two points in the past 6 months, without mucopurulent bloody stool; ④ Presence of IBS-like symptoms met the Rome IV diagnostic criteria for at least 6 months. More detailed information about inclusion and exclusion criteria could be seen in supplementary methods. All patients signed an informed consent form and completed a questionnaire. The flow chart of the study could be seen in Supplementary Fig. 1.

### Sample collection

All participants followed a light, low-residue diet for at least three days before sample collection. The subjects who need to take colonoscopy provided samples on the day before the bowel preparation. Please see supplementary method for further details.

### Lipid extraction and analysis

Samples were extracted using the MTBE method [21, 22]. Eighty μL of precooled methanol and 400 μL of MTBE were added to 50 μL of serum, 100 mg of feces or 5 mg of intestinal tissue. The process of mixing, centrifugation, nitrogen drying and redissolution was carried out.

Untargeted lipid metabolite analysis was performed using ultra-performance liquid chromatography-high resolution mass spectrometry (UPLC-HRMS) on a Vanquish Flex system coupled to an Orbitrap ID-X system (Thermo Fisher™ Scientific, Waltham, MA, USA) and ACQUITY UPLC CSH Phenyl-Hexyl Column (1.7 µm, 2.1 mm × 100 mm, Waters™, Manitowish Waters, WI, USA) at 40 °C. Mobile phase A was 10 mM ammonium formate and 0.1% formic acid in acetonitrile:water (60:40, v/v), and mobile phase B was 10 mM ammonium formate and 0.1% formic acid in isopropanol:acetonitrile (90:10, v/v) [22, 23].

Analysis on polyunsaturated fatty acids (PUFAs) was using ultra-performance liquid chromatography tandem triple quadrupole mass spectrometry (UPLC-QqQ MS) conducted on a Vanquish Flex system coupled to a TSQ AltisTM (Thermo ScientificTM, Waltham, MA, USA) and ACQUITY UPLC BEH C18 Column (1.7 µm, 2.1 mm × 100 mm, WatersTM, Manitowish Waters, WI, USA) at 45 °C. Mobile phase A was 15 mM ammonium formate in wate, and mobile phase B was methanol. Please see supplementary methods and supplementary table 1-3 for further details.

### PUFAs and omega-6/omega-3 PUFA Ratio

Linoleic acid (LA; 18:2n-6) is the shorter chain n-6 precursor of arachidonic acid (ARA; 20:4n-6). Alpha-linlenic acid (ALA; 18:3n-3) is the shorter chain n-3 PUFA precursor of longer chain eicosapentaenoic acid (EPA; 20:5n-3) and docosahexaenoic acid (DHA; 22:6n-3). The concentration of 5 PUFAs were determined by external standard with 1.0–1000 μg/L matrix matched standard solutions. Our analysis of PUFA ratios focuses on the long-chain PUFAs, calculated as AA/(EPA + DHA).

### Data processing and statistical analysis

The raw data were processed using Progenesis QI software (Waters Corporation). Retention time (RT) between 1.5 to 9.1 min were retained, and the percentage of metabolites non-detected rate in real samples exceeded 15%, or if the relative standard deviation (RSD) value exceeded 30%, the metabolites would be excluded from subsequent statistical analyses. The metabolites were annotated using the HMDB, KEGG, and LIPID MAPS databases. The peak areas of single lipid species were normalized by the sum of the peak areas of all detected lipid species. Statistical analyses were performed using R (version 4.1.2) and SPSS version 22.0 software (SPSS Inc., USA). The Pearson correlation coefficients between QC samples were calculated based on the normalized quantitative values of metabolites. We performed principal component analysis (PCA), partial least square discriminant analysis (PLS-DA), and orthogonal partial least squares discriminant analysis (OPLS-DA) with the variables scaled, and the importance of variables in the projection (VIP) scores was calculated from the PLS-DA model. Between-group differences were analyzed by linear regression after adjusting for age, sex, and BMI. The log 2 value was used to normalize the quantitative values after adding a small pseudo count as the dependent variable, and age, sex, and BMI were used as dependent variables for the study group (with the IBS-D group used as the reference). The coefficients associated with the study group were interpreted as the log2-fold change (FC). To compare the UCR-IBS group with the HC group, an additional linear regression was similarly performed using data from the two study groups. Q-values were estimated from P values for false discovery rate control. Significant differences were filtered using the criteria of FC > 1.5 or FC < 0.667, *Q* value < 0.05, and VIP > 1.0.

## Results

### Baseline characteristics of the participants

A total of 95 participants were recruited; there were 35 participants in the HC group, 39 in the IBS-D group, and 21 in the UCR-IBS group. The patients’ baseline characteristics are described in Table 1*.* There were no significant differences in demographic characteristics among the three groups. The IBS-SSS scores were not different between the IBS-D and UCR-IBS groups, with half of the patients being in moderate condition. The diseases groups had higher scores for anxiety and depression, which suggests the role of psychologic factors in chronic intestinal symptoms. They also had worse quality of sleep than the HC group (*P* = 0.001). There were no differences in dietary habits among the three groups, except that the IBS-D and UCR-IBS groups tended to abstain from spicy foods (Supplementary Table 4).

**Table 1:** Baseline demographic and clinical characteristics

Name	HC *N* = 35	IBS-D *N* = 39	UCR-IBS *N* = 21	*P* value
Age, mean (SD), years	32.34 ± 8.82	35.77 ± 10.80	39.30 ± 11.12	0.052
Sex (male, %)				0.057
Male	14 (40.0)	26 (66.7)	13 (61.9)	
Female	21 (60.0)	13 (33.3)	8 (38.1)	
BMI, kg/m^2^	21.82 ± 3.31	23.17 ± 2.99	22.84 ± 3.46	0.077
Education level, no. (%)				0.332
Primary school	0 (0)	1 (2.6)	0 (0)	
Junior school	2 (5.7)	1 (2.9)	0 (0)	
High school	0 (0)	4 (10.3)	1 (0)	
College	7 (20.0)	9 (23.1)	8 (38.1)	
Graduate	26 (74.3)	24 (61.5)	12 (57.1)	
Income status, no. (%), ¥				0.306
< 300,00	3 (8.6)	1 (2.6)	0 (0.0)	
300,000–1500,000	11 (31.4)	13 (33.3)	5 (23.8)	
1500,000–3000,000	12 (34.3)	9 (23.1)	10 (47.6)	
> 3000,000	9 (25.7)	16 (41.0)	6 (28.6)	
Alcohol consumption, no. (%)				0.112
Never	27 (77.1)	22 (56.4)	13 (61.9)	
Sometimes	6 (17.1)	15 (38.5)	6 (28.6)	
Always	0 (0.0)	2 (5.1)	0 (0.0)	
Have quit	2 (5.7)	0 (0)	2 (9.5)	
Cigarette smoking, no. (%)				0.072
Never smoker	30 (85.7)	33 (84.6)	16 (76.2)	
< 10/d	1 (2.9)	5 (12.8)	1 (4.8)	
10–20/d	2 (5.7)	0 (0)	0 (0)	
Past smoker	2 (5.7)	1 (2.6)	4 (19.0)	
Exercise, no. (%)				0.693
Never	3 (8.6)	2 (5.1)	1 (4.8)	
Sometimes	26 (74.3)	28 (71.8)	13 (61.9)	
Always	6 (17.1)	9 (23.1)	7 (33.3)	
HADS scores, mean (SD), years				
Anxiety	4.74 ± 2.49	5.54 ± 3.98	5.71 ± 3.78	0.353
Depression	4.20 ± 2.42	4.36 ± 3.55	4.62 ± 4.23	0.903
PSQI scores, mean (SD), years	8.77 ± 3.45	13.90 ± 6.56	13.86 ± 7.88	0.001
Course of disease, mean (SD), years	na	4.32 ± 3.46	6.33 ± 4.41	
Extent of colitis, no. (%)				
Ulcerative proctitis	na	na	15 (71.5)	
Left-sided ulcerative colitis	na	na	4 (19.0)	
Extensive ulcerative colitis	na	na	2 (9.5)	
IBS-SSS scores, mean (SD), years	25.71 ± 18.68	202.56 ± 72.73	188.10 ± 66.90	< 0.001
Mild (75–174)	na	10 (25.6)	6 (28.6)	
Moderate (175–299)	na	25 (64.1)	13 (61.9)	
Severe (≥ 300)	na	4 (10.3)	2 (9.5)	

### Comprehensive overview of lipidomic findings

Valid samples were included in the analysis as follows: 85 serum samples (26 from HC, 38 from IBS-D, 21 from UCR-IBS), 87 stool samples (31 from HC, 38 from IBS-D, 18 from UCR-IBS), and 38 intestinal mucosal tissues (17 from HC, 14 from IBS-D, 7 from UCR-IBS). Ultimately, 3869 colon biopsy metabolite signals, 2063 serum metabolite signals, and 3067 fecal metabolite signals were detected in both positive and negative modes. There were 76 lipids with significant changes in the mucosa sample, 345 in the feces sample and 120 in the serum. Among these lipids, fatty acyls (FAs) were enriched in every sample, and glycerophospholipids (GPs), glycerolipids (GLs), and sphingolipids (SLs) also accounted for a significant proportion (Supplementary Table 5).

### Detailed analysis of lipid profiles in intestinal mucosa biopsies

**Fig. 1:**
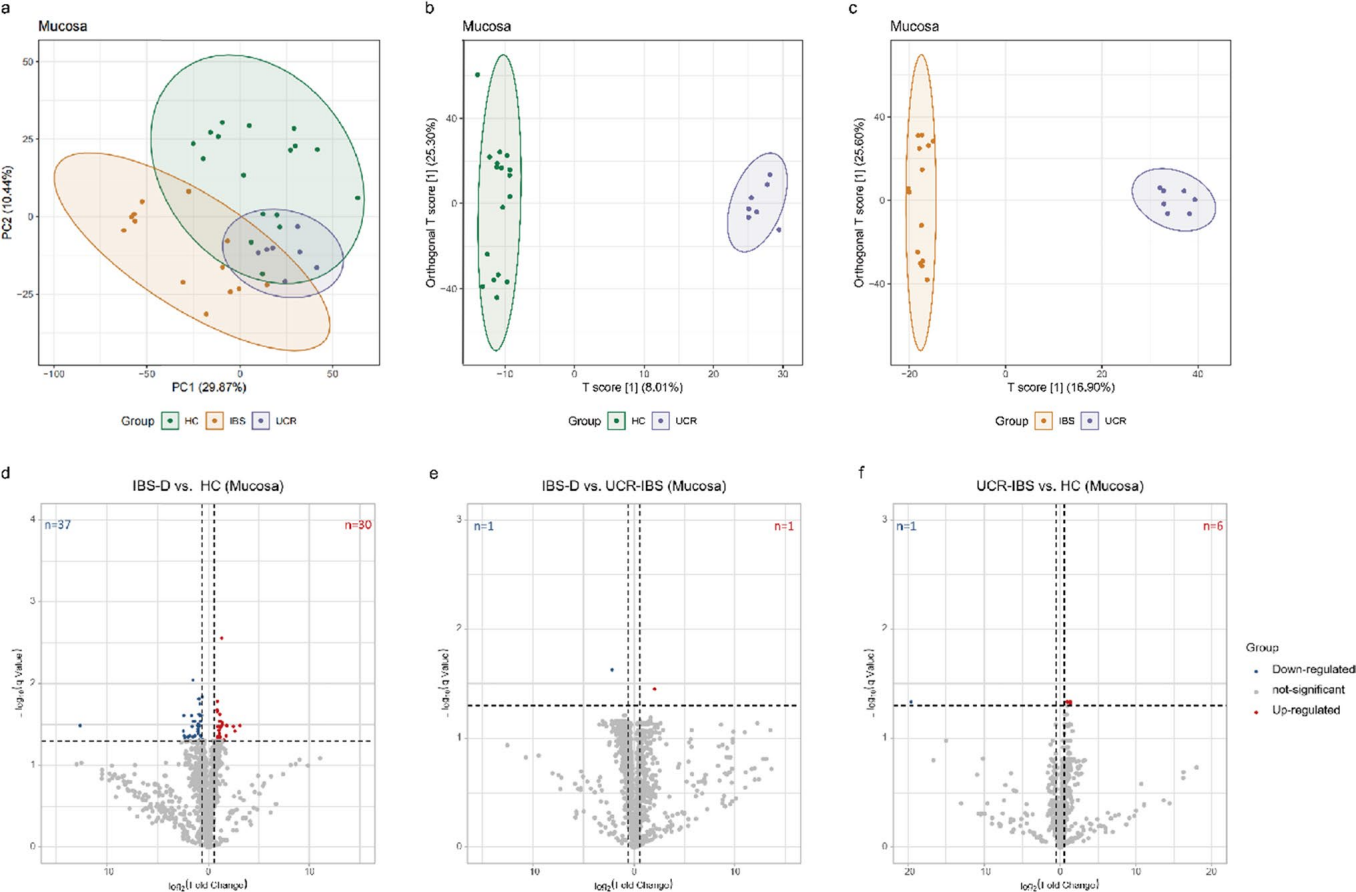
Multivariate analysis of the mucosal lipid profiles and volcano plots. **a** PLS-DA score plots. PC1 and PC2 were 29.87% and 10.44%. **b** OPLS-DA score plots of the HC and UCR-IBS groups, with R2X 0.501 cum, R2Y 0.993 cum, Q2 0.58. **c** OPLS-DA score plots of the IBS-D and UCR-IBS groups, with R2X 0.571 cum, R2Y 0.994 cum, Q2 0.583. **d**–**f** Differential volcano plots for the three comparisons. The blue dots represent downregulated genes, the red dots represent upregulated genes, and the gray dots represent genes whose expression did not significantly change

Mucosal lipid profiles in colon biopsies were assessed to determine significant changes in lipid composition in the IBS-D and UCR-IBS groups compared with the control group. We employed PLS-DA to reveal the separation (Fig. 1a). The results suggested that while there was some separation among the HC, IBS-D, and UCR-IBS groups based on the first two principal components, the distinction was not clear. The HC group was somewhat separated from the IBS-D group, but the UCR-IBS group was similar to both the HC and IBS-D groups. Thus, OPLS-DA was applied for better separation, and in this model, the metabolic profiles of the UCR-IBS vs. HC and IBS-D groups differed significantly (Fig. 1b and c). Specifically, in the comparison between the IBS-D and HC groups, 67 lipids were altered, with 37 showing an increase in relative concentration (Fig. 1d). The significant changes in lipid levels between the IBS-D and HC groups suggested that pronounced dysregulation of lipid metabolism in IBS-D patients may be involved in gastrointestinal dysfunction. In contrast, only two lipid changes were observed between the IBS-D and UCR-IBS groups. PE (24:0/22:2(13Z,16Z)) increased by 4.77-fold, and LysoPC (16:1(9Z)) decreased (Fig. 1e). In addition, seven lipids displayed differences between the UCR-IBS and HC groups, and PS (O-16:0/15:1(9Z)) was significantly decreased by 27.11-fold (Fig. 1f).

### Co-occurrence distribution of lipid species in the IBS-D and UCR-IBS groups

**Fig. 2:**
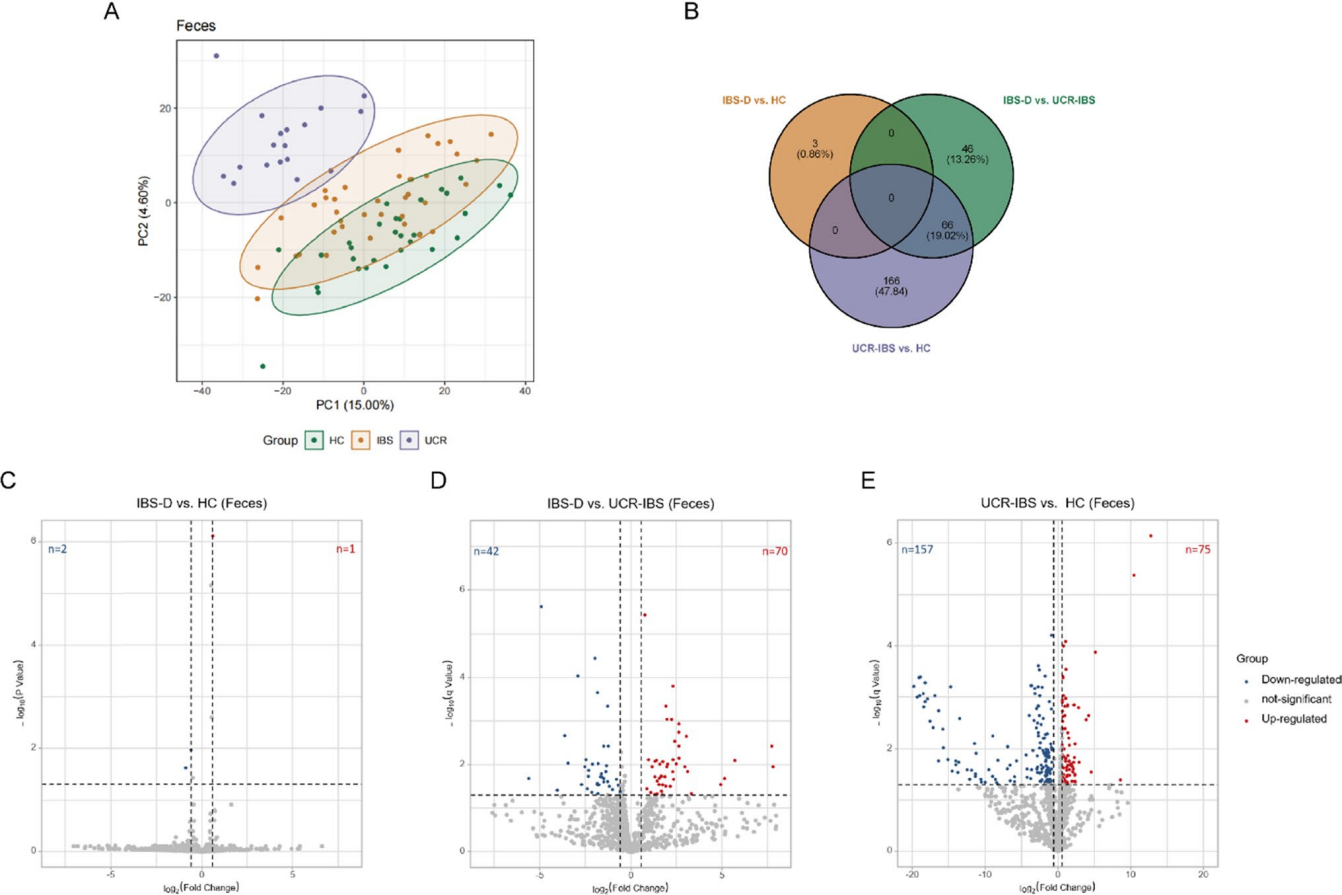
Multivariate analysis of the fecal lipid profiles. **a** PLS-DA score plots. PC1 and PC2 were 15.00% and 4.60%. **b** Venn diagrams of feces samples showing the number of overlapping lipids in the IBS-D, UCR-IBS, and HC groups. **c**–**e** Differential volcano plots for the three comparisons. The blue dots represent downregulated genes, the red dots represent upregulated genes, and the gray dots represent genes whose expression did not significantly change

Lipids in the feces samples changed the most. Among the altered lipid classes, GPs, FAs, GLs and SPs were the four most varied classes (Supplementary Table 5). PLS-DA clearly distinguished the HC group from both the IBS-D and UCR-IBS groups along PC1. The IBS-D and UCR-IBS groups showed slight overlap but were primarily separated along PC2 (Fig. 2a). The fecal lipidome revealed a significant overlap between the IBS-D vs. UCR-IBS and UCR-IBS vs. HC comparisons (Fig. 2b), with 66 (38.3%) lipids overlapping, including classes such as GPs, FAs, and SPs. Specifically, among these overlapping lipids, 45 (68.2%) were increased in the IBS-D vs. UCR-IBS group. Of these increased lipids, most were GPs and FAs. However, the overlapping lipids demonstrating the largest change were increased in the UCR-IBS vs. HC group, as these were increased by 53- to 1851-fold. Thus, the lipid changes in fecal samples were more significant between the UCR-IBS group and the HC group. Only 19 STs were identified in feces, including 3 bile acids. The STs were generally decreased in the UCR-IBS vs. HC group. Compared to that in the HC group, the level of 2,2-dibromoacetic acid, a type of fatty acid, was increased in the IBS-D group (Fig. 2c). The majority of lipids were decreased in the UCR-IBS vs. HC group, whereas over half of the lipids were increased in the IBS-D vs. UCR-IBS group (Fig. 2d and e).

### Systematic description of serum lipid distribution

**Fig. 3:**
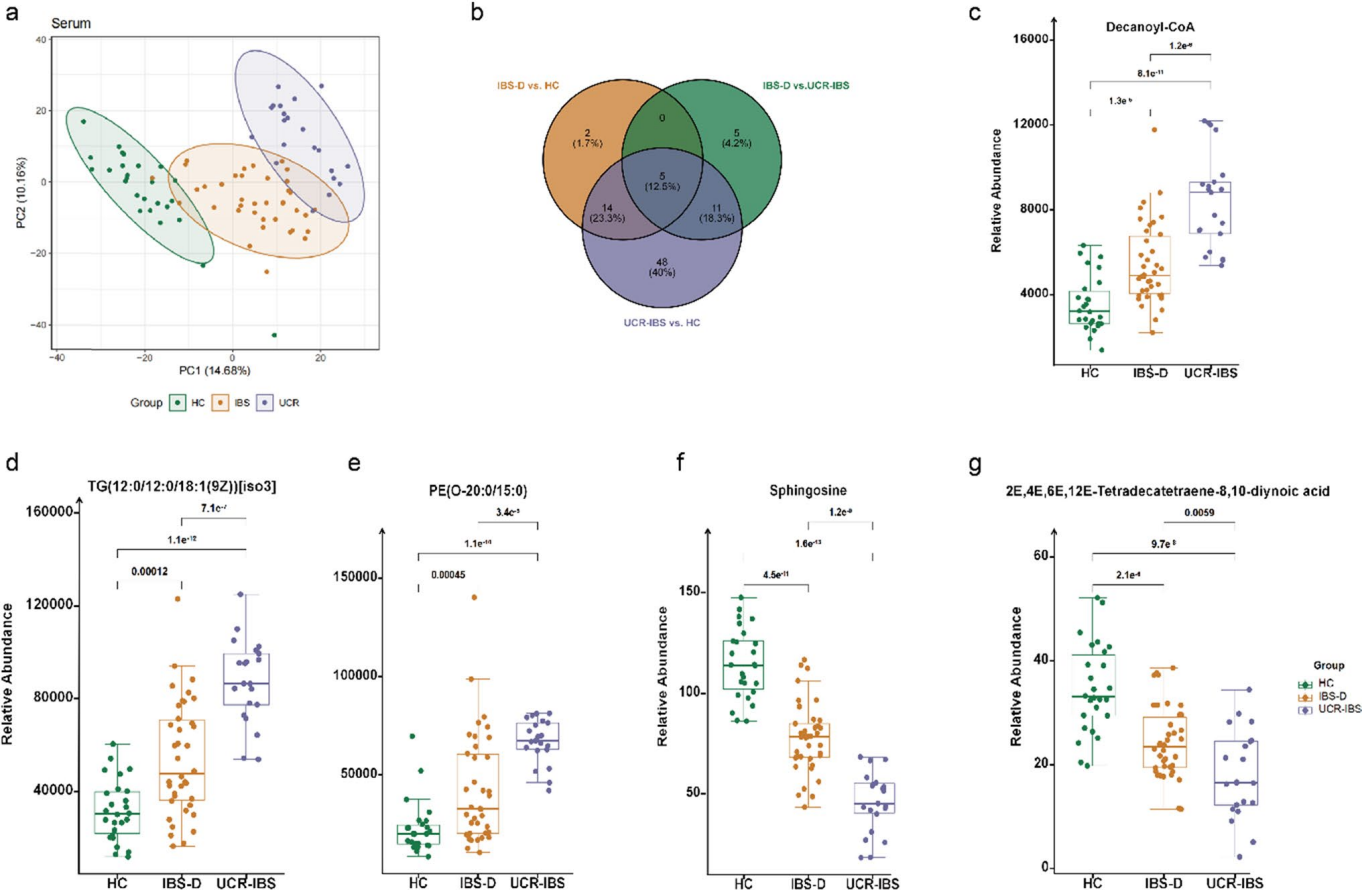
Lipid profiles in serum samples. **a** PLS-DA score plots. PC1 and PC2 were 14.68% and 10.16%. **b** Venn diagrams of serum samples showing the number of overlapping lipids in the IBS-D, UCR-IBS, and HC groups. **c**–**g** The relative abundances of the overlapping lipids. All five lipids were significantly different among the three groups, with *P* < 0.05

There was a significant difference in serum lipid profiles between the HC group and the others (Fig. 3a). The IBS-D and UCR-IBS groups overlapped somewhat, suggesting similarities in their lipid profiles in systemic circulation. In addition, 5 (12.5%) lipids, including decanoyl-CoA, 2E,4E,6E,12E-tetradecatetraene-8,10-diynoic acid, PE(O-20:0/15:0), TG (12:0/12:0/18:1(9Z)) [iso3], and sphingosine, were common in all three comparisons (Fig. 3b). The relative abundances of decanoyl-CoA, TG (12:0/12:0/18:1(9Z)) [iso3] and PE(O-20:0/15:0) were greatest in the UCR-IBS group, followed by those in the IBS-D group, with *P* < 0.01 (Fig. 3c–e). In contrast, 2E,4E,6E,12E-tetradecatetraene-8,10-diynoic acid (Fig. 3f) and sphingosine (Fig. 3g) were lowest in the UCR-IBS group and significantly highest in the HC group. Considering the characteristics of the three groups, these five lipids may indicate differences between functional and organic diseases.

### Analysis and interpretation of the enriched metabolic pathways

**Fig. 4:**
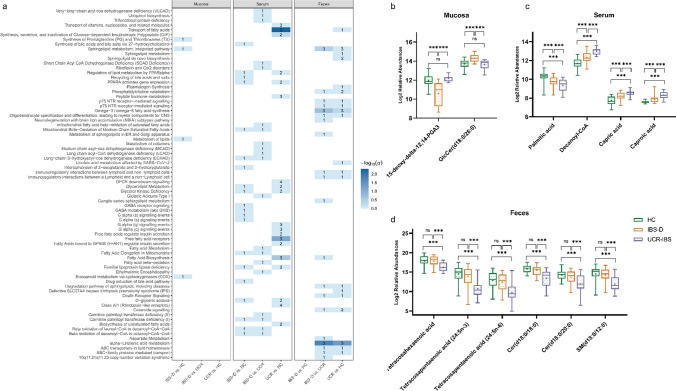
Heatmap and box plots of metabolic pathways. **a** Lipidomic enrichment analysis across multiple biologic matrices. Lipid pathways in mucosa, serum, and fecal samples were compared among the IBS-D, UCR-IBS and HC groups. The y-axis shows the annotation of each pathway, and the color intensity corresponds to the -log10 *q*-value, with darker cells indicating higher enrichment levels. Numbers within the heatmap denote hit lipids. **b**–**d** Relative abundances of lipids involved in metabolic pathways in the three groups (***, *P* < 0.01; ns, no significance)

Lipid pathway enrichment analysis of the identified lipids was performed with MetaboAnalyst 6.0. Comprehensive analysis revealed distinct metabolic pathway engagement across mucosa, serum, and feces samples (Fig. 4a). The heatmap provides a visual representation of the pathways that exhibited significant alterations among the tested conditions. In the mucosa samples, only the IBS-D group vs. the HC group comparison exhibited changes in lipid metabolism pathways associated with 15-deoxy-delta-12,14-PGA2 and GlcCer (d18:0/26:0). 15-Deoxy-delta-12,14-PGA2 was significantly lower in the IBS-D group than in the UCR-IBS and HC groups, and GlcCer (d18:0/26:0) was significantly greater (Fig. 4b). However, the q-value of these pathways did not indicate a statistically significant difference. In serum samples, there were notable differences in pathways such as the 'transport of fatty acids' and ‘free fatty acids receptor’ between the UCR-IBS and IBS-D groups. The involved lipids were capric acid, palmitic acid, caproic acid, and decanoyl-CoA. Compared to IBS-D patients, UCR-IBS patients showed significantly elevated levels of capric acid, caproic acid, and decanoyl-CoA. In contrast, palmitic acid was lower in UCR-IBS patients than in IBS-D patients (Fig. 4c). The fecal samples exhibited distinct patterns of pathway engagement, with alpha-linolenic acid metabolism, omega-3/omega-6 fatty acid synthesis, and sphingolipid metabolism showing significant differences between the IBS-D and UCR groups and between the UCR-IBS and HC groups. All lipids involved in the above pathways were significantly decreased in the feces samples of the UCR-IBS group (Fig. 4d).

### Imbalance of omega-6/omega-3 PUFAs ratio in serum

Based on the findings from untargeted lipidomics, we observed significant enrichment of metabolites in the alpha-linolenic acid metabolic pathway. In order to verify the repeatability and reliability of the results, we conducted targeted reanalysis of PUFA substances in the serum samples. Our analysis revealed a notable decrease in DHA levels in the IBS-D and UCR-IBS groups compared to the HC group, with statistically significant differences between the UCR-IBS and HC groups. No significant differences were detected in ALA and EPA levels across the three groups. Furthermore, the omega-6 PUFA levels were significantly elevated in both the IBS-D and UCR-IBS groups, though no significant difference was observed between them. Importantly, the omega-6/omega-3 PUFA ratio was significantly higher in the IBS-D and UCR-IBS groups compared to the HC group (Table 2).

**Table 2:** Analysis of PUFAs in serum

	HC *N* = 26	IBS-D *N* = 38	UCR-IBS *N* = 21	*P* value
ALA, μmol/L	10.11 ± 3.41	10.36 ± 2.79	10.18 ± 3.83	0.950
EPA, μmol/L	0.30 ± 0.07	0.32 ± 0.06	0.32 ± 0.08	0.393
DHA, μmol/L	2.72 ± 0.93	2.27 ± 1.08	2.058 ± 0.66^†^	0.047
LA, μmol/L	48.62 ± 29.87	88.07 ± 28.63^*^	81.67 ± 26.52^†^	< 0.001
ARA, μmol/L	2.78 ± 0.82	3.99 ± 1.17^*^	3.70 ± 0.84^†^	< 0.001
Omega-6/omega-3 PUFA ratio^§^	1.00 ± 0.39	1.69 ± 0.61^*^	1.66 ± 0.53^†^	< 0.001

^*^HC vs. IBS-D group, *P* < 0.05

^†^HC vs. UCR-IBS group, *P* < 0.05

^§^Computed as AA/(DHA + EPA)

## Discussion

IBS and IBD have similar symptoms and internal relationships, but the pathologic mechanisms underlying these relationships remain elusive. Lipid metabolism plays a critical role in human function, especially in biologic signaling and the regulation of inflammation. With the emergence of new technologies, lipids can be analyzed extensively. Many studies have focused on the metabolome of IBS and IBD patients and have shown that metabolites change in different disease conditions [19, 20]. Previous studies have also shown that fatty acid metabolism plays an important role by participating in the inflammatory pathway [16, 24]. However, few studies have described lipidomic analyses of multiple biologic matrices. In addition, compared with those of the intestinal mucosa and serum, stool samples might be more useful for reflecting the state of the disease and revealing its intrinsic pathogenesis. In this study, we targeted the lipidome of IBS-D and UCR-IBS patients to describe comprehensive lipid profiles by analyzing multiple biologic samples, and found the abnormal expression of PUFAs in intestinal diseases.

In mucosa samples, the lipid analysis indicated subtle differences among IBS-D patients, UCR-IBS patients, and healthy control individuals. The significant increase in PE (24:0/22:2(13Z,16Z)) and decrease in LysoPC(16:1(9Z)) in IBS-D patients compared to UCR-IBS patients suggest that these lipids may play critical roles in the pathophysiology of these conditions. PE (24:0/22:2(13Z,16Z)) is a kind of phosphatidylethanolamine that has many different combinations of fatty acids of varying lengths and saturation. It is involved in cellular membrane structure and signaling, potentially affecting intestinal barrier function or inflammation in the intestinal mucosa [25, 26]. Diab et al. [17] reported that a kind of PE (PE (38:3)) changed the most in the mucosa of UC patients. This PE increased under active conditions and decreased in remission, suggesting that this kind of lipid could be used to monitor the development of UC. In our study, PE was increased in IBS-D patients, which may reflect the slight inflammation in IBS-D patients compared to UCR patients. Lysophosphatidylcholines (LysoPCs), such as LysoPC(16:1(9Z)), are also involved in inflammatory processes [27]. The dramatic 27.11-fold decrease in PS(O-16:0/15:1) in UCR-IBS patients compared to healthy control individuals could indicate significant disruptions in cellular signaling and membrane composition, as PS plays a key role in cell apoptosis, coagulation, and immune response regulation [28]. Such a marked reduction might reflect changes in intestinal epithelial cell turnover, barrier function, or immune responses.

The distinct lipid profiles in serum, particularly those of the five overlapping lipids, point to specific metabolic disruptions in UCR-IBS. The elevated levels of decanoyl-CoA, PE(O-20:0/15:0), and TG (12:0/12:0/18:1(9Z)) [iso3] in UCR-IBS patients suggest a distinct metabolic pattern that might influence the disease pathophysiology. Decanoyl-CoA, a kind of saturated fatty acyl CoA, is involved in fatty acid metabolism and oxidation, indicating altered energy utilization or storage [13]. PE(O-20:0/15:0) is another phosphatidylethanolamine that is similar to PE (24:0/22:2(13Z,16Z)) and is detected in mucosa samples. TG (12:0/12:0/18:1(9Z)) [iso3], a triglyceride, has been reported to be elevated in UCR patients compared to healthy control individuals [29, 30], suggesting that this difference may be due to complex interactions between inflammatory cytokines. In our study, we also found the highest TG level in the UCR-IBS group, which is consistent with previous studies. Sphingosine, a long-chain unsaturated amino alcohol, acts as an endogenous inducer of apoptosis by inhibiting cell proliferation and promoting programmed cell death [31]. The most important metabolite of sphingosine is its phosphorylation product in the first step, which is sphingosine-1-phosphate (S1P). The role of S1P in immune cell trafficking has been widely studied [32], and S1P is also a new therapeutic target for reducing inflammation in IBD patients [33]. Our study found reduced levels of sphingosine in the IBS-D and UCR-IBS groups; however, we did not directly measure S1P levels. In addition, the samples we analyzed were from patients with UC in remission. Therefore, the specific roles and associations of sphingosine and S1P in these conditions require further investigation.

Another interesting finding is the differences among different biologic samples. Among the three specimens, feces contained the most lipid species, and mucosa samples contained the fewest. Feces are very important samples in gastroenterology, not only because they are convenient to obtain but also, they are directly collected from the intestines. Thus, they would be more comprehensively reflect the metabolic activities of both microbes and the body [34]. For the mucosa, we tried to collect representative regions, but only very small biopsies could be obtained, representing more localized and specific profiles of the intestinal tissue. In addition, the sample size is also the smallest among the three samples. All of these factors could explain why the mucosa samples had the fewest differential substances. In intestinal mucosa samples, the greatest changes were observed between the IBS-D and HC groups, and the other group comparisons showed few differences. This may imply that the mucosa samples of the IBS-D and UCR-IBS groups are possibly similar. However, the opposite results were obtained for the fecal samples. Taking the characteristics of the two kinds of specimens into account, the intestinal tissue is better to reflect the local metabolic condition of the intestine, but the feces could better represent the whole gut metabolites. Considering the convenience of sample collection and its effectiveness in reflecting metabolic information, fecal samples might have advantages in lipidomic studies.

Notably, we found that the fatty acid metabolism pathways, especially alpha-linolenic acid metabolism and omega-3/omega-6 fatty acid synthesis, were significantly enriched in fecal and serum samples in the IBS-D and UCR-IBS groups, which is consistent with our previous study [16]. ALA is a polyunsaturated fatty acid that is essential in the human diet. It is the substrate for the synthesis of EPA, DHA and other anti-inflammatory omega-3 PUFAs, which are important for human functions [35]. From the untargeted lipidomics results, tetracosahexaenoic acid (THA), tetracosapentaenoic acid (24:5n-6) and tetracosapentaenoic acid (24:5n-3), very important precursors of DHA in the omega-3 PUFA biosynthetic process [36, 37], are significantly changed. The reduction of precursors affects the synthesis of downstream substances, resulting in an imbalance in the omega-6/omega-3 ratio. We also verified this by retesting the PUFAs in serum samples, showing the consistency with untargeted lipidomics results. DHA can interfere with a variety of inflammatory signaling pathways, regulating and reducing inflammatory responses. A reduction in DHA reflects an imbalance between anti-inflammatory and proinflammatory mediators (e.g., ARA). In addition, a high ratio of omega-6/omega-3 PUFAs predicts various aspects of psychologic distress, including the onset of post-partum depression and mood disorders [38, 39]. This imbalance is more pronounced in IBS patients, leading to more severe somatic disorders and depressive symptoms [40]. In our study, DHA were significantly decreased in the UCR-IBS group, while LA, ARA and the omega-6/omega-3 PUFAs ratio were significantly higher in both UCR-IBS and IBS-D group. The imbalances in the PUFAs indicated the IBS-D and UCR-IBS might share the same mechanism in psychologic tissues and the activation of inflammatory pathways.

There were some limitations in our study. First, the sample size was relatively small, and we did not match the case and control groups. Nevertheless, we adjusted for these variables during the statistical analysis process to minimize the potential impact on the results. Second, we didnot provide standard diet for participants. But we required them follow a light, low-residue diet for at least 3 days before sample collection to reduce differences from dietary factors as long as possible. Besides, only a portion of the subjects underwent endoscopic screening in this study. However, the remainder of UCR-IBS group had the colonoscopy within a year, and we used fecal calprotectin with blood inflammation markers to screen them out and ensure them in remission. Furthermore, given the varying sampling preferences for IBS and UC observed in prior studies [19, 20], with IBS in sigmoid and UC in rectum, we chose to collect mucosal samples from the rectosigmoid junction. This site could provide a relatively comprehensive analysis of the metabolic condition both in sigmoid and rectum.

In conclusion, this study is the first to comprehensively describe the distribution and differences in the lipidome of IBS-D patients, UCR-IBS patients via an integrated multisample analysis of intestinal mucosa, feces, and serum samples. We found that fatty acids, particularly PUFAs, participate in the IBS and IBD-IBS processes, suggesting that the dysregulation of omega-3 and omega-6 metabolism is associated with psychologic disorders and inflammatory activation. We also revealed that fecal samples were more likely to reflect the condition more comprehensively than serum or intestinal mucosa samples.

## Supplementary Information

Below is the link to the electronic supplementary material.

Supplementary file1 (DOCX 230 KB)

Supplementary file2 (DOCX 23 KB)
