# Supplementary material for: Multisample lipidomic profiles of irritable bowel syndrome and irritable bowel syndrome-like symptoms in patients with inflammatory bowel disease: new insight into the recognition of the same symptoms in different diseases
Source: J Gastroenterol. 2024 Sep 10;59(11):1000–10. doi: 10.1007/s00535-024-02148-1 (PMC11496327; doi:10.1007/s00535-024-02148-1)
Supplement: Supplementary file 1 — Supplementary file1 (DOCX 230 KB) [file 535_2024_2148_MOESM1_ESM.docx]

**Supplementary Table 1.** Polyunsaturated fatty acids with corresponding SRM transitions (quadrupole 1 m/z, quadrupole 3 m/z, collision energy, RF lens, and retention time)

| Analyte | Q1 (m/z) | Q3 (m/z) | Collision Energy (eV) | RF lens  (V) | Retention time (min) |
| --- | --- | --- | --- | --- | --- |
| α-Linolenic acid | 277.2 | 259.1 | 18 | 55 | 4.00 |
| Docosahexaenoic acid | 327.3 | 283.3 | 10 | 80 | 4.19 |
| Eicosapentaenoic acid | 301.1 | 257.2 | 10 | 60 | 3.98 |
| α-Linoleic acid | 279.2 | 261.3 | 18 | 65 | 4.28 |
| Arachidonic acid | 303.2 | 259.3 | 13 | 70 | 4.25 |

**Supplementary Table 2.** Polyunsaturated fatty acids with their class, limit of detection (LOD), and limit of quantitation (LOQ)

| Analyte | Class | Limit of Detection^1^  (ng/mL) | Limit of Quantitation^2^  (ng/mL) |
| --- | --- | --- | --- |
| α-*L*inolenic acid | Omega-3 | 0.94 | 3.12 |
| Docosahexaenoic acid | Omega-3 | 0.05 | 0.17 |
| Eicosapentaenoic acid | Omega-3 | 0.02 | 0.08 |
| α-Linoleic acid | Omega-6 | 1.13 | 3.75 |
| Arachidonic acid | Omega-6 | 0.11 | 0.37 |

^1^ The method detection limit of PUFA was determined by a signal-to-noise ratio (S/N) of 3:1.

^2^ Limit of quantitation was determined by S/N of 10:1.

**Supplementary Table 3.** Recoveries, intraday and interday precision in serum

| Analyte | Precision (RSD, %) | | Recoveries (%) | | |
| --- | --- | --- | --- | --- | --- |
|  | Intra-day | Inter-day | Low | Medium | High |
| α-Linolenic acid | 1.72 | 1.22 | 87.16 | 82.03 | 90.71 |
| Docosahexaenoic acid | 1.66 | 1.09 | 93.97 | 86.71 | 90.52 |
| Eicosapentaenoic acid | 6.29 | 1.71 | 114.42 | 108.89 | 119.63 |
| α-Linoleic acid | 2.12 | 0.62 | 92.00 | 88.95 | 100.19 |
| Arachidonic acid | 3.57 | 0.97 | 80.11 | 85.83 | 89.56 |

**Supplementary Table 4.** Dietary characteristics of subjects

|  | HC  N=35 | IBS-D  N=39 | UCR-IBS  N=21 | p value |
| --- | --- | --- | --- | --- |
| Staple food, No. (%) |  |  |  | 0.170 |
| rice | 25(71.4) | 26(66.7) | 9(42.9) |  |
| cooked wheaten food | 9(25.7) | 9(23.1) | 10(47.6) |  |
| Roughage | 1(2.9) | 4(10.3) | 2(9.5) |  |
| Daily diet composition, No. (%) |  |  |  | 0.472 |
| Almost Staple food | 7(20.0) | 4(10.3) | 2(9.5) |  |
| Almost dishes | 7(20.0) | 11(28.2) | 3(14.3) |  |
| Two both | 21(60.0) | 24(61.5) | 16(76.2) |  |
| Intake of dishes, No. (%) |  |  |  | 0.163 |
| Vegetarianism | 5(14.3) | 8(20.5) | 5(23.8) |  |
| Meat-based | 12(34.3) | 10(25.6) | 1(4.8) |  |
| Normal | 18(51.4) | 21(53.8) | 15(71.4) |  |
| Intake of fruits, No. (%) |  |  |  | 0.118 |
| Hardly | 5(14.3) | 3(7.7) | 2(9.5) |  |
| Little (<200g/d) | 20(57.1) | 24(61.5) | 6(28.6) |  |
| Moderate (200-350g/d) | 9(25.7) | 11(28.2) | 13(61.9) |  |
| Exceeding (>350g/d) | 1(2.9) | 1(2.6) | 0(0) |  |
| Intake of vegetables, No. (%) |  |  |  | 0.268 |
| Hardly | 1(2.9) | 2(5.1) | 0(0) |  |
| Little (<300g/d) | 15(42.9) | 13(33.3) | 3(14.3) |  |
| Moderate (300-400g/d) | 18(51.4) | 23(59.0) | 16(76.2) |  |
| Exceeding (>400g/d) | 1(2.9) | 1(2.6) | 2(9.5) |  |
| Intake of dairy products, No. (%) |  |  |  | 0.503 |
| Hardly | 6(17.1) | 10(25.6) | 3(14.3) |  |
| Little (<200ml/d) | 16(45.7) | 15(38.5) | 8(38.1) |  |
| Moderate (200-300ml/d) | 13(37.1) | 12(30.8) | 10(47.6) |  |
| Exceeding (>300ml/d) | 0(0) | 2(5.1) | 0(0) |  |
| Intake of soy products and nuts, No. (%) |  |  |  | 0.113 |
| Hardly | 9(25.7) | 6(15.4) | 5(23.8) |  |
| Little (<20g/d) | 19(54.3) | 23(59.0) | 5(23.8) |  |
| Moderate (20-35g/d) | 7(20.0) | 9(23.1) | 10(47.6) |  |
| Exceeding (>35g/d) | 0(0) | 1(2.6) | 1(4.8) |  |
| Intake of meats, No. (%) |  |  |  | 0.587 |
| Hardly | 0(0) | 0(0) | 0(0) |  |
| Little (<40g/d) | 4(11.4) | 4(10.3) | 4(19.0) |  |
| Moderate (40-75g/d) | 25(71.4) | 24(61.5) | 14(66.7) |  |
| Exceeding (>75g/d) | 6(17.1) | 11(28.2) | 3(14.3) |  |
| Intake of aquatic product, No. (%) |  |  |  | 0.471 |
| Hardly | 8(22.9) | 10(25.6) | 8(38.1) |  |
| Little (<40g/d) | 20(57.1) | 21(53.8) | 8(38.1) |  |
| Moderate (40-75g/d) | 7(20.0) | 6(15.4) | 5(23.8) |  |
| Exceeding (>75g/d) | 0(0) | 2(5.1) | 0(0) |  |
| Intake of spicy food, No. (%) |  |  |  | 0.016 |
| Hardly | 7(20.0) | 15(38.5) | 13(61.9) |  |
| Sometimes | 20(57.1) | 21(53.8) | 6(28.6) |  |
| Always | 8(22.9) | 3(7.7) | 2(9.5) |  |
| Intake of greasy food, No. (%) |  |  |  | 0.361 |
| Hardly | 8(22.9) | 12(30.8) | 6(28.6) |  |
| Sometimes | 23(65.7) | 21(53.8) | 15(71.4) |  |
| Always | 4(11.4) | 6(15.4) | 0(0) |  |
| Intake of raw food, No. (%) |  |  |  | 0.596 |
| Hardly | 24(68.6) | 26(66.7) | 17(81.0) |  |
| Sometimes | 8(22.9) | 11(28.2) | 4(19.0) |  |
| Always | 3(8.6) | 2(5.1) | 0(0) |  |
| Intake of sodas, No. (%) |  |  |  | 0.475 |
| Hardly | 18(51.4) | 22(56.4) | 15(71.4) |  |
| Sometimes | 14(40.0) | 13(33.3) | 6(28.6) |  |
| Always | 3(8.6) | 4(10.3) | 0(0) |  |
| Intake of coffee, No. (%) |  |  |  | 0.382 |
| Hardly | 25(71.4) | 27(69.2) | 16(76.2) |  |
| Sometimes | 8(22.9) | 5(12.8) | 2(9.5) |  |
| Always | 2(5.7) | 7(17.9) | 3(14.3) |  |
| Intake of strong tea, No. (%) |  |  |  | 0.775 |
| Hardly | 22(62.9) | 23(59.0) | 14(66.7) |  |
| Sometimes | 8(22.9) | 13(33.3) | 5(23.8) |  |
| Always | 5(14.3) | 3(7.7) | 2(9.5) |  |

**Supplementary Table 5.** Overview of lipid changes in the IBS-D, UCR-IBS, and HC groups.

| Name | Mucosa | Feces | Serum |
| --- | --- | --- | --- |
| Significant number of metabolites | 76 | 347 | 120 |
| Group Comparison  (Significant/Increased number of metabolites) |  |  |  |
| IBS vs. HC | 67/37 | 3/1 | 21/8 |
| IBS vs. UCR-IBS | 2/1 | 112/70 | 21/10 |
| UCR-IBS vs. HC | 7/6 | 232/75 | 78/37 |
| Lipid Class, No. (%) |  |  |  |
| Fatty acyls (FAs) | 22(28.95) | 81(23.34) | 62(51.67) |
| Glycerolipids (GLs) | 5(6.58) | 73(21.04) | 22(18.33) |
| Glycerophospholipids (GPs) | 27(35.53) | 108(31.12) | 15(12.50) |
| Prenol lipids (PRs) | 6(7.89) | 7(2.02) | 2(1.67) |
| Sphingolipids (SPs) | 9(11.84) | 59(17.00) | 7(5.83) |
| Steroids and steroid derivatives (STs) | 7(9.21) | 19(5.48) | 12(10.00) |


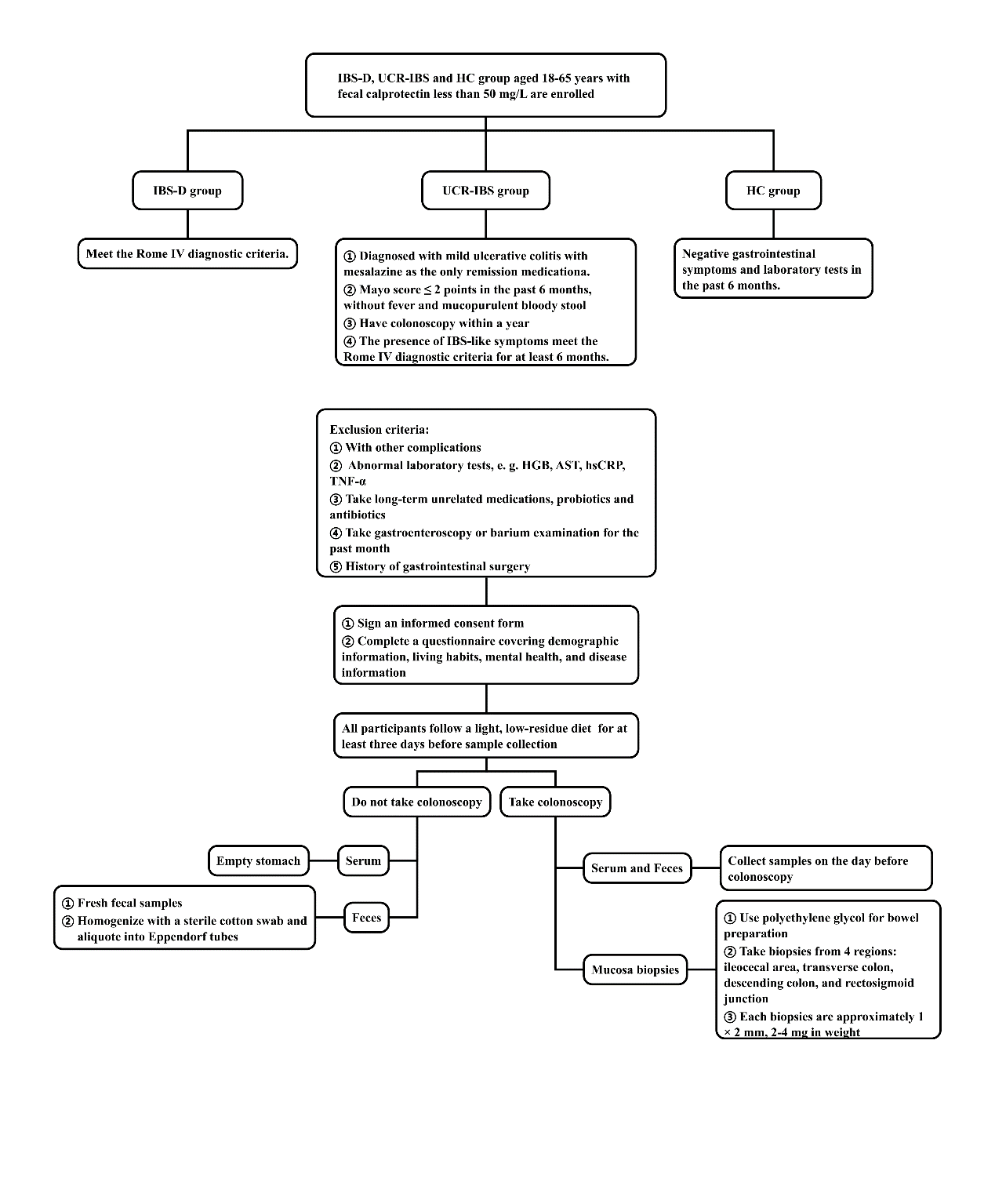
 **Supplementary Fig. 1** The flowchart of the study.

**
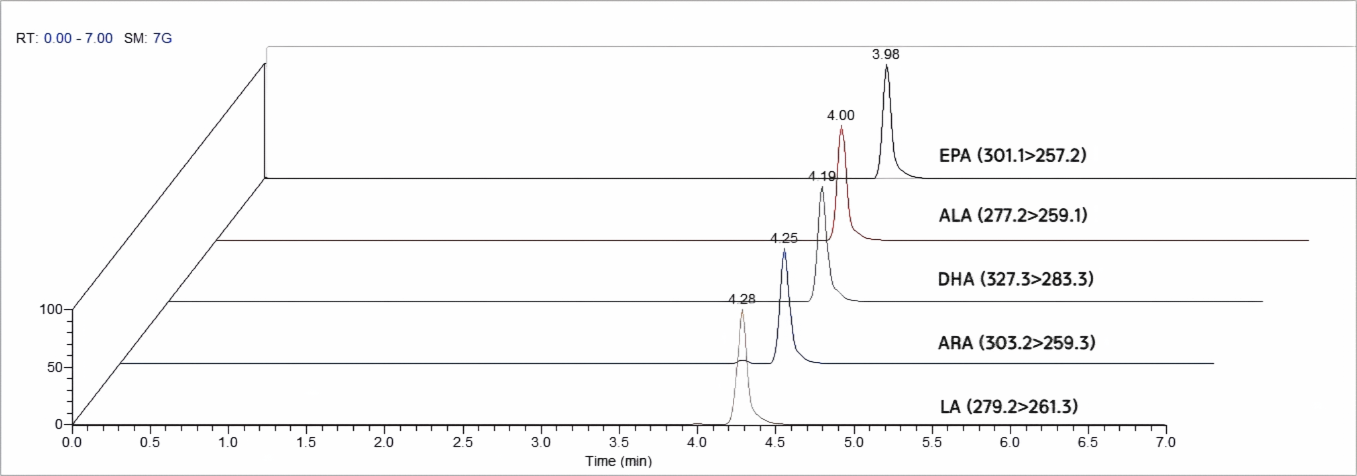
**

**Supplementary Fig. 2** Chromatogram of the 5 standards of analysis ordered by retention times (in brackets, transitions of precursor and quantifier ions (m/z)).
