# Supplementary material for: Multisample lipidomic profiles of irritable bowel syndrome and irritable bowel syndrome-like symptoms in patients with inflammatory bowel disease: new insight into the recognition of the same symptoms in different diseases
Source: J Gastroenterol. 2024 Sep 10;59(11):1000–10. doi: 10.1007/s00535-024-02148-1 (PMC11496327; doi:10.1007/s00535-024-02148-1)
Supplement: Supplementary file 2 — Supplementary file2 (DOCX 23 KB) [file 535_2024_2148_MOESM2_ESM.docx]

**Multisample lipidomic profiles of irritable bowel syndrome and irritable bowel syndrome-like symptoms in patients with inflammatory bowel disease: new insight into the recognition of the same symptoms in different diseases**

Guorong Chen^1#^, Xuan Wu^2,3#^, Huiting Zhu^4^, Kemin Li^4^, Junhai Zhang^1^, Shijie Sun^4^, Huifen Wang^4^, Miao Wang^4^, Bing Shao^3^, Hui Li^3^, Yanli Zhang^4*^, Shiyu Du^1*^

**Affiliations**

^1^Department of Gastroenterology, China-Japan Friendship Hospital (Institute of Clinical Medical Sciences), Peking Union Medical College & Chinese Academy of Medical Sciences, Beijing, 100029, China

^2^School of Public Health, Capital Medical University, Beijing 100069, China

^3^Beijing Key Laboratory of Diagnostic and Traceability Technologies for Food Poisoning, Beijing Center for Disease Prevention and Control, Beijing 100013, China

^4^Department of Gastroenterology, China-Japan Friendship Hospital, Beijing 100029, China

**Methods**

***Design***

This single-center, case‒control study was conducted from September 2022 to August 2023 in the Department of Gastroenterology in China-Japan Friendship Hospital, Beijing, China. This study was approved by the Ethics Committee of China-Japan Friendship Hospital (2022-KY-023) and registered on the Chinese Clinical Trial Registry website (ChiCTR2200060224).

***Participants***

This study included three groups: the HC group, the IBS-D group, and the UCR-IBS group. All participants were aged 18-65 years with a level of fecal calprotectin less than 50 mg/L. Patients in the IBS-D group met the Rome IV diagnostic criteria. Patients in the UCR-IBS group should meet the following criteria: ① Diagnosed with mild ulcerative colitis, with mesalazine used as the remission medication; ② Have colonoscopy within a year; ③ Mayo score ≤ 2 points in the past 6 months, especially without fever and mucopurulent bloody stool; ④ Presence of IBS-like symptoms met the Rome IV diagnostic criteria for at least 6 months. Healthy individuals had no positive gastrointestinal symptoms and findings in laboratory tests in the past 6 months. The exclusion criteria were as followed: ① With other complications; ② Abnormal laboratory test (e.g. CRP, TNF-α) within 6 months; ③ Take long-term unrelated medications, probiotics, and antibiotics; ④ Take gastroenteroscopy or barium examination for the past month; ⑤ History of gastrointestinal surgery. All patients signed an informed consent form and completed a questionnaire covering demographic information, living habits, mental health, and disease information. The full flow chart of the study could be seen in Supplementary Fig. 1.

***Sample collection***

All participants followed a light, low-residue diet for at least three days before sample collection. The subjects who need to take colonoscopy provided samples on the day before the bowel preparation. A total of 5 mL of fasting blood was collected and centrifuged at 4000 rpm and 4°C for 10 min to obtain the serum. Fresh fecal samples were homogenized with a sterile cotton swab and then aliquoted into Eppendorf tubes. Colonoscopies were performed as needed or upon subject consent. All participants used polyethylene glycol for bowel preparation. Biopsies (each sample was approximately 1 × 2 mm, 5-10 mg in weight) were taken from four regions without obvious lesions—the ileocecal area, transverse colon, descending colon, and junction of rectum and sigmoid colon—and placed into different Eppendorf tubes. All the samples were flash-frozen in liquid nitrogen for at least 30 min and then quickly transferred to a -80°C freezer until further analysis.

***Lipid extraction***

For lipidomic analysis, samples were extracted using the MTBE method. Briefly, 80 μL of precooled methanol and 400 μL of MTBE were added to 50 μL of serum, 100 mg of feces or 5 mg of intestinal tissue. The samples were ultrasonically extracted for 15 min, after which 100 μL of ultrapure water was added to form a two-phase layered solution. Then, the samples were centrifuged at 4°C and 14000 rpm for 20 min, after which the supernatant was allowed to dry under nitrogen. The extraction process was repeated twice. Then, the extracts were redissolved in 200 μL of a methanol:isopropanol solution (50:50, v/v, 0.1% formic acid, 10 mM ammonium acetate), and the supernatant was removed for subsequent analysis. Quality control (QC) samples were prepared by mixing equal volumes of the reconstitution solution from all samples.

***Untargeted lipid analysis***

Untargeted lipid metabolite analysis was performed using ultra-performance liquid chromatography-high resolution mass spectrometry (UPLC-HRMS) on a Vanquish Flex system coupled to an Orbitrap ID-X system (Thermo Fisher^TM^ Scientific, Waltham, MA, USA) and ACQUITY UPLC CSH Phenyl-Hexyl Column (1.7 µm, 2.1 mm x 100 mm, Waters^TM^, Manitowish Waters, WI, USA) at 40°C. Mobile phase A was 10 mM ammonium formate and 0.1% formic acid in acetonitrile:water (60:40, v/v), and mobile phase B was 10 mM ammonium formate and 0.1% formic acid in isopropanol:acetonitrile (90:10, v/v). The optimized gradient elution process was as follows: 0-1.5 min, 50% B; 1.5-9.0 min, 50-85% B; 9.0-9.1 min, 85-100% B; 9.1-11.0 min, 100% B; 11.0-11.1 min, 100-50% B; and 11.1-13 min, 50% B. The flow rate was set at 0.30 mL/min, and the injected volume was 5 μL. The data were acquired from *m/z* 100-1000 Da at a heated electrospray ionization source (ESI) in the positive and negative ionization modes. Low-energy data were acquired at a collision energy of 10 eV, and high-energy data were acquired at a collision energy of 30−45 eV. The operating parameters were as follows: sheath gas pressure, 40 arbitrary units (arb); auxiliary gas pressure, 10 (arb); capillary temperature, 400°C; full MS (resolution 120,000); MS/MS (resolution 17,500); and spray voltage, 3.8 kV (positive) or −3.0 kV (negative).

***Targeted lipid analysis***

Analysis on polyunsaturated fatty acids (PUFAs) was using ultra-performance liquid chromatography tandem triple quadrupole mass spectrometry (UPLC-QqQ MS) conducted on a Vanquish Flex system coupled to a TSQ Altis^TM^ (Thermo Scientific^TM^, Waltham, MA, USA) and ACQUITY UPLC BEH C18 Column (1.7 µm, 2.1 mm x 100 mm, Waters^TM^, Manitowish Waters, WI, USA) at 45°C. Mobile phase A was 15 mM ammonium formate in wate, and mobile phase B was methanol. The optimized gradient elution process was as follows: 0-1.0 min, 50% B; 1.0-1.01 min, 50-90% B; 1.01-6 min, 90-100% B; 6.0-7.0 min, 100% B; 7.0-7.01 min, 100-50% B; and 7.01-8 min, 50% B. The flow rate was set at 0.30 mL/min, and the injected volume was 5 μL. Selected reaction monitoring was performed in negative electrospray ionization mode. The transitions are provided in Supplementary Table 1. The source conditions were as follows: electrspray voltage 3200 V, sheath gas 50 units, auxiliary gas 15 units, ion transfer tube temp 325℃，and vaporizer temp 350℃. Analytes with their class, chromatogram, limit of detection (LOD), limit of quantitation (LOQ), recoveries, intraday and interday precision are reported in Supplementary Table 2-3 and Fig. 2.

***PUFAs and n-6/n-3 PUFA Ratio***

Linoleic acid (LA; 18:2n-6) is the shorter chain n-6 precursor of arachidonic acid (ARA; 20:4n-6). Alpha-linlenic acid (ALA; 18:3n-3) is the shorter chain n-3 PUFA precursor of longer chain eicosapentaenoic acid (EPA; 20:5n-3) and docosahexaenoic acid (DHA; 22:6n-3). The concentration of 5 PUFAs were determined by external standard with 1.0 to 1000 μg/L matrix matched standard solutions. Our analysis of PUFA ratios focuses on the long-chain PUFAs, calculated as AA/(EPA+DHA).

***Data processing and statistical analysis***

The untargeted lipidomic raw data were processed using Progenesis QI software (Waters Corporation), including initial data processing, feature detection, alignment, normalization, statistical analysis, and annotation of metabolites. Retention time (RT) of each metabolite between 1.5 to 9.1 min were retained, and the percentage of metabolites non-detected rate in real samples exceeded 15%, or if the relative standard deviation (RSD) value exceeded 30%, which was calculated for each metabolite based on QC samples, the metabolites would be excluded from subsequent statistical analyses. The metabolites were annotated using the HMDB, KEGG, and LIPID MAPS databases. The peak areas of single lipid species were normalized by the sum of the peak areas of all detected lipid species. Hence, the quantitative data for each lipid species were expressed as the relative concentration to the total amount of lipids.

Statistical analyses were performed using R (version 4.1.2) and SPSS version 22.0 software (SPSS Inc., USA). The Pearson correlation coefficients between QC samples were calculated based on the normalized quantitative values of metabolites. We performed principal component analysis (PCA), partial least square discriminant analysis (PLS-DA), and orthogonal partial least squares discriminant analysis (OPLS-DA) with the variables scaled, and the importance of variables in the projection (VIP) scores was calculated from the PLS-DA model using the R package 'RVAideMemoire'. The first two principal components (PCs) in the results were plotted, with the color denoting the study group, and ellipses were used to visualize the data dispersion and identify potential clusters or trends. For each metabolite, between-group differences were analyzed by linear regression after adjusting for confounders, including age, sex, and BMI. The log 2 value was used to normalize the quantitative values after adding a small pseudo count as the dependent variable, and age, sex, and BMI were used as dependent variables for the study group (with the IBS-D group used as the reference). The coefficients associated with the study group were interpreted as the log2-fold change (FC). To compare the UCR-IBS group with the HC group, an additional linear regression was similarly performed using data from the two study groups. Q-values were estimated from p values for false discovery rate control. Significant differences were filtered using the criteria of FC > 1.5 or FC < 0.667, Q-value < 0.05, and VIP > 1.0.
